# Host and symbiont genetic contributions to fitness in a *Trichogramma–Wolbachia* symbiosis

**DOI:** 10.7717/peerj.4655

**Published:** 2018-04-19

**Authors:** James E. Russell, Leonard Nunney, Michael Saum, Richard Stouthamer

**Affiliations:** 1 School of Science and Technology, Georgia Gwinnett College, Lawrenceville, GA, USA; 2 Department of Biology, University of California, Riverside, CA, USA; 3 Department of Entomology, University of California, Riverside, CA, USA

**Keywords:** *Wolbachia*, Sex ratio, *Trichogramma*, Parthenogenesis, Fitness

## Abstract

The fitness effects associated with *Wolbachia* infection have wide-ranging ecological and evolutionary consequences for host species. How these effects are modulated by the relative influence of host and *Wolbachia* genomes has been described as a balancing act of genomic cooperation and conflict. For vertically transmitted symbionts, like cytoplasmic *Wolbachia*, concordant host–symbiont fitness interests would seem to select for genomic cooperation. However, *Wolbachia*’s ability to manipulate host reproductive systems and distort offspring sex ratios presents an evolutionary conflict of interest with infected hosts. In the parthenogenesis-inducing (PI) form of *Wolbachia* found in many haplodiploid insects, *Wolbachia* fitness is realized through females and is enhanced by their feminization of male embryos and subsequent parthenogenetic reproduction. In contrast, as long as *Wolbachia* is not fixed in a population and sexual reproduction persists, fitness for the host species is realized through both male and female offspring production. How these cooperating and competing interests interact and the relative influence of host and *Wolbachia* genomes were investigated in the egg parasitoid *Trichogramma kaykai*, where *Wolbachia* infection has remained at a low frequency in the field. A factorial design in which laboratory cultures of *Wolbachia*-infected *T. kaykai* were cured and re-infected with alternative *Wolbachia* strains was used to determine the relative influence of host and *Wolbachia* genomes on host fitness values. Our results suggest fitness variation is largely a function of host genetic background, except in the case of offspring sex ratio where a significant interaction between host and *Wolbachia* genomes was found. We also find a significant effect associated with the horizontal transfer of *Wolbachia* strains, which we discuss in terms of the potential for coadaptation in PI-*Wolbachia* symbioses.

## Introduction

The alphaproteobacterium *Wolbachia pipientis* is a widely distributed cytoplasmic symbiont among arthropods and nematodes, and occupies an ecological niche distinguished by manipulation of host reproduction. For many arthropod species host reproduction is manipulated in a parasitic manner that involves distortion of host sex ratios by male-killing, feminization or parthenogenesis-induction, and by creating reproductive barriers between infected and uninfected individuals via a process referred to as cytoplasmic incompatibility (Werren, 1997). *Wolbachia* is also considered a mutualistic obligate symbiont among nematodes where host fertility appears to be dependent upon *Wolbachia* infection (Pfarr & Hoerauf, 2007). The various reproductive effects associated with *Wolbachia* make it one of the most influential bacterial infections known with regard to the its effect on the fitness of infected host populations.

Fitness for vertically transmitted *Wolbachia* is ultimately linked to female host fitness since the primary mode of transmission for *Wolbachia* is maternal inheritance (*Wolbachia* resides in the cytoplasm of host reproductive cells). Hence, the effects of *Wolbachia* infections on different aspects of host fitness directly affect the nature of the symbioses between *Wolbachia* and their hosts: negative fitness effects resulting in host–symbiont conflicts if they enhance *Wolbachia* fitness (e.g., by increasing transmission rate); and positive fitness effects resulting in host–symbiont cooperation. For long-term coevolved symbioses theoretical predictions and empirical results suggest selection will favor alignment of fitness interests for maternally inherited symbionts and their female hosts (Turelli, 1994; Herre et al., 1999; Wade & Goodnight, 2006; Weeks et al., 2007). However, the ability to manipulate host reproductive systems by sex ratio distortion presents a potential source of conflict between *Wolbachia* symbionts and their hosts, since the fitness interests of sexually reproducing hosts differ from maternally inherited *Wolbachia* symbionts regarding offspring sex ratios, with sexual hosts gaining optimum fitness through male and female offspring and *Wolbachia* gaining optimum fitness through female offspring alone.

The parthenogenesis-inducing (PI) form of *Wolbachia* found in several haplodiploid arthropods feminizes male embryos allowing infected females to produce female offspring without mating (Stouthamer, Luck & Hamilton, 1990). The mechanism by which *Wolbachia* induces parthenogenesis varies among host species from a mechanism called gamete duplication (*Trichogramma kaykai* and other species) to a mechanism resembling apomixis in *Bryobia* mite species (Stouthamer & Kazmer, 1994; Weeks & Breeuwer, 2001); however, in either case the end result is the parthenogenetic production of infected diploid females from unfertilized eggs. PI-*Wolbachia* infection frequencies in various species and populations of wasps, thrips, and mites are extremely high (Arakaki, Miyoshi & Noda, 2001; Weeks & Breeuwer, 2001; Huigens & Stouthamer, 2003; Pannebakker et al., 2005; Kremer et al., 2009). Though the PI form of *Wolbachia* does not directly prevent fertilization and sexual reproduction, in populations fixed for infection, females have lost the ability to fertilize eggs and reproduce sexually (Pannebakker et al., 2005). As a result, such populations (and some entire species) are dependent on *Wolbachia* for reproduction (Russell & Stouthamer, 2011; Stouthamer et al., 2010).

*Trichogramma kaykai*, a minute egg parasitoid of the Mojave Desert, is one of the rare examples of a species with a low frequency PI-*Wolbachia* infection. The infection frequency has been repeatedly measured over the past 20 years with observed frequencies reaching no higher than 26% infected females (Stouthamer & Kazmer, 1994; Stouthamer et al., 2001; Huigens, 2003; Russell, 2008). Unlike populations fixed for PI-*Wolbachia*, infected *T. kaykai* females are capable of sexual *and* parthenogenetic reproduction. Infected *T. kaykai* females mate with uninfected males at high frequencies and high levels of heterozygosity have been observed within the infected *T. kaykai* population (Huigens, 2003; Russell, 2008).

Given the unusual characteristics of the *T. kaykai–Wolbachia* symbiosis, namely, a low infection frequency and facultative parthenogenesis, the fitness effects of associated with *Wolbachia* infection in *T. kaykai* have been the subject of numerous studies (Huigens et al., 2004; Miura & Tagami, 2004; Tagami, Miura & Stouthamer, 2001; Russell et al., 2016). Comparisons between *Wolbachia*-infected and antibiotically cured *T. kaykai* have shown consistent negative fitness effects associated with *Wolbachia* infection. *Wolbachia*-free treatments produce more offspring and are less likely to die in pupal development (Tagami, Miura & Stouthamer, 2001; Huigens et al., 2004; Russell et al., 2016). *Wolbachia*-free treatments also fertilize eggs and produce offspring sex ratios at frequencies similar to what is observed in the uninfected population (Tagami, Miura & Stouthamer, 2002; Russell et al., 2016). The fitness costs associated with infection may be a factor in the observed low infection frequency in the field, however, host limitation may mitigate the fitness costs associated with infection (Lindsey & Stouthamer, 2017a). How the fitness costs associated with infection are attributed to host, *Wolbachia*, and/or host–*Wolbachia* interactions is currently unknown for *T. kaykai*.

**Figure 1:**
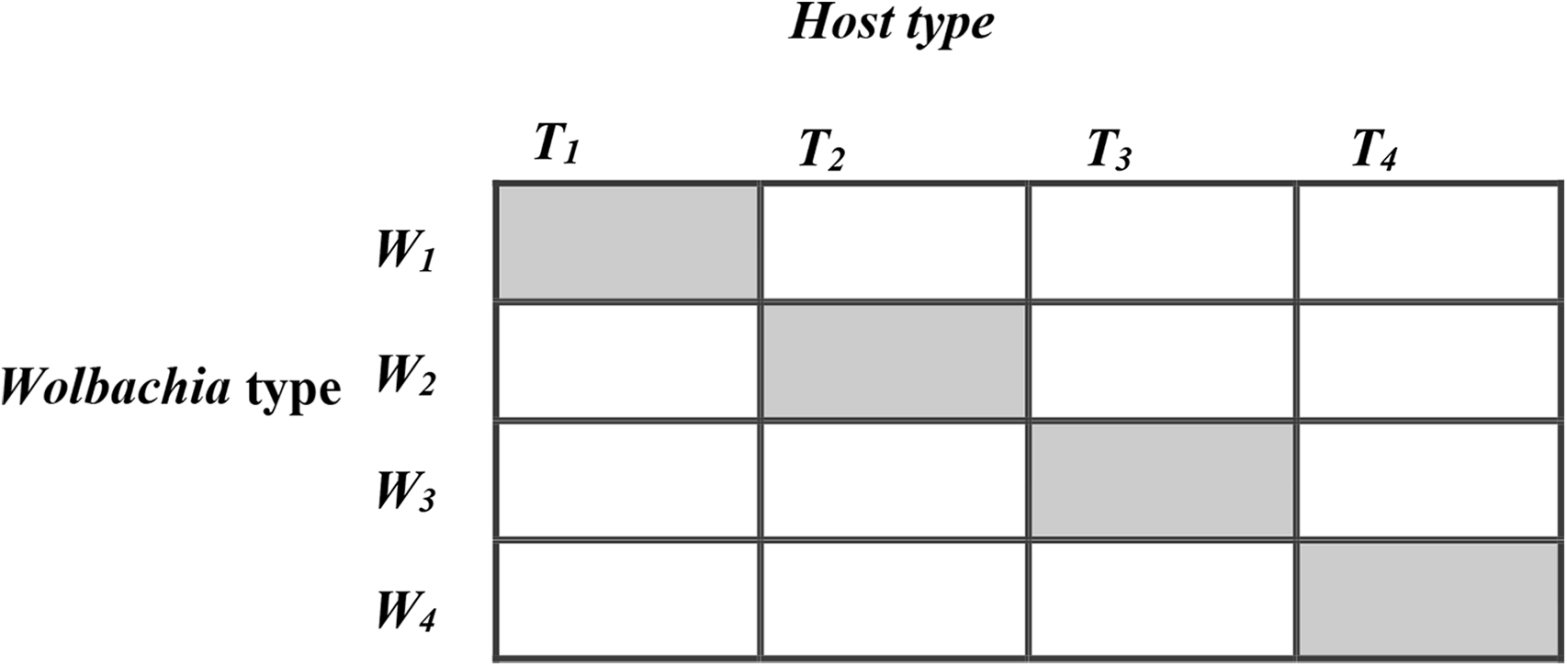
The 4 × 4 factorial design for horizontal transfer of *Wolbachia* in Host *Trichogramma kaykai*. The shaded diagonal represents the original infections from field-caught *T. kaykai* females; all off-diagonals represent novel infections created by horizontal transfer of *Wolbachia*.

To investigate these relative fitness effects of host *T. kaykai* genetic background, *Wolbachia* background, and horizontal transfer of *Wolbachia*, we used a curing/re-infection protocol. Using four clonal lines of field-caught infected *T. kaykai* that were maintained separately in the laboratory over 97 generations, we created a factorial design of the four *T. kaykai* (*T_i_*) and *Wolbachia* (*W_i_*) genomes (Fig. 1) to test hypotheses related to the *T. kaykai–Wolbachia* interaction. We addressed two main questions. First, since novel (*not* strictly co-inherited) cyto-nuclear combinations are typical in the field in this species, do such combinations show fitness variation and, if so, is it driven by differences among host genomes or symbiont genomes, or by their combination? Second, does horizontal transfer of PI-*Wolbachia* result in decreased fitness?

## Materials and Methods

In this study we utilized a horizontal transmission technique (Huigens et al., 2000) to factorially combine the nuclear and *Wolbachia* genomes in four isofemale *T. kaykai* lines, enabling us to partition the relative fitness effects of *T. kaykai* (host) genome, *Wolbachia* genome, and the host/*Wolbachia* interaction. Most PI-*Wolbachia*-infected species are dependent on *Wolbachia* for reproduction and cannot reproduce sexually; thus cured cultures cannot be established. The *T. kaykai* system is unique among the PI-*Wolbachia*-infected species in that cured sexual cultures can be established and re-infected.

### Insect cultures: *T. kaykai*

*Trichogramma kaykai* is a small hymenopteran egg parasitoid distributed throughout the Mojave Desert of southern California (Pinto, Stouthamer & Platner, 1997). The *Wolbachia*-infected *T. kaykai* isofemale lines used in this study were collected from two locations. The lines *T_1_W_1_* and *T_2_W_2_* (where *T* and *W* refer to the *T. kaykai* and *Wolbachia* genomes, respectively) were collected on April 25, 2004 and May 5, 2005, respectively, off Kelbaker Road in the Kelso Dunes region of the Mojave National Preserve (34°57′55.31″N, 115°38′35.75″W). Collection permits were obtained from Debra Hughson (science advisor, Mojave National Preserve, permit number: moja-2006-sci-0015) and annual reports contributed to the United States National Park Service, Mojave National Preserve. The lines *T_3_W_3_* and *T_4_W_4_* were collected on June 14, 2003 off California State Highway 247 near the Stoddard Ridge (34°37′48.52″N, 116°57′08.99″W); no specific permission was required for collection at this location. For both locations, eggs of the butterfly *Apodemia mormo* (Riodinidae) were removed from the host plant *Eriogonum inflatum* (Polygonaceae), taken to the lab, and placed individually in 1.2 ml collection vials. The vials were incubated at 24 °C, L:D = 16:8 h and 50% relative humidity until emergence. Parasitized *A. mormo* eggs typically contain between three and five *Trichogramma* wasps. Several *Trichogramma* species have been collected from *A. mormo* eggs in the Mojave Desert, but the dominant egg parasitoid during the time of our collection was *T. kaykai*. *T. kaykai* species were identified morphologically (Pinto, Stouthamer & Platner, 1997). Upon emergence, broods containing male wasps were discarded, since they were presumed uninfected. Females from all female broods were isolated for further testing. No field collections involved handling endangered or protected species.

### *Wolbachia* infection status

The infection status of the isolated wasps was first established by virgin production of daughters and confirmed by amplifying 16S rDNA characteristic of *Wolbachia*. To produce offspring, the isolated virgin females were given *Ephestia kuehniella* host eggs attached to cardstock paper with double-sided tape (eggcards). If the resulting F1 offspring were mostly female the line was maintained in the laboratory as an infected isofemale culture. All lines used in this study produced highly female-biased offspring sex ratios as virgins, producing some males as they aged. This is a pattern typical of *Wolbachia*-infected *T. kaykai* females (Hohmann, Luck & Stouthamer, 2001; Miura & Tagami, 2004). Consistent with this parthenogenesis, the lines also tested positive for *Wolbachia* infection, based on 16S rDNA. The 16S rDNA was amplified using PCR primers specific for *Wolbachia:* 16sBf 5′-TTCGGCCGGATTTTACACAA-3′, 16sBr 5′-AGGGATTAGCTTAGGCTTG-3′ (Werren, Windsor & Guo, 1995), using a thermocycling protocol of: initial denaturation at 94 °C for 2 min, followed by 38 cycles at 94 °C for 30 s, 55 °C for 50 s, 72 °C for 1 min 30 s; and a final extension at 72 °C for 10 min. Four *Wolbachia* MLST loci were used for typing the strains used in this study (Baldo et al., 2006). No variation among the *Wolbachia* types from the four host lines was observed for these four loci. The *coxA*, *gatB*, and *hcpA* MLST loci in *T. kaykai* matched strain type 486 in the *Wolbachia* MLST database. There was no strain type match for the *T. kaykai fbpA* locus; this locus shared sequence similarity with *fbpA* sequence 359, with four sequence differences.

### Curing of infected lines

The two experimental lines, *T_1_W_1_*, *T_2_W_2_*, were maintained in laboratory cultures for 138 generations and the two experimental lines, *T_3_W_3_*, *T_4_W_4_*, were maintained for 97 generations after initial isofemale isolation from field collected samples. After this period each of the lines was subdivided into an infected and a cured culture. Cured cultures were obtained following three generations of antibiotic treatment. This involved feeding newly emerged infected females a 5 mg/ml rifampcin/honey solution for one day. After feeding, females in the curing protocol were given eggcards and the rifampcin/honey mixture. The emergent second and third generations were likewise given rifampcin/honey solution for one day prior to eggcards and oviposition. The following (fourth) generation pupae were isolated and emerging females given *E. kuehniella* host eggs for virgin oviposition on day 1. On day 2 the previous day’s host eggs were removed and placed in a 12 × 75 mm glass culture tube for rearing in order to confirm the all-male offspring expected following curing. A male from the same line and an eggcard were placed in the culture tube containing the isolated female, and this culture was then maintained as a cured sexual line. After three generations of mass mating, pupae from the cured population were isolated and female offspring were tested again for cured status by the failure to amplify *Wolbachia*16S rDNA, and the virgin production of only male offspring.

### Horizontal transfer of *Wolbachia*

Using the four experimental lines, novel combinations were created by horizontal transfer in the laboratory by infection of each cured subline with the three alternative types of *Wolbachia*. A superparasitization technique developed by Huigens et al. (2000) was used to horizontally transfer *Wolbachia* from the original infected cultures into cured sublines to create novel *T. kaykai/Wolbachia* combinations. All original lines were uniquely genotyped by observable agarose gel band size differences using microsatellite markers specifically designed for *T. kaykai* (Huigens, 2003) to confirm successful horizontal transfer of *Wolbachia*. Cured and infected females were allowed to oviposit on the same host (*Trichoplusia ni*). F1 females from superparasitized hosts were genotyped and tested for *Wolbachia* infection status. Successful horizontal transfer of *Wolbachia* infection was determined by production of female offspring by virgin females who were identified by their nuclear genotype as having come from cured sublines, thus creating new cross-infected isofemale cultures. The original lines were not re-created by re-infection of cured lines. All cross-infected lines (novel infections created by horizontal transfer of *Wolbachia*) were cultured for 20 generations prior to final fitness testing. The 20 generation period was chosen to: (1) assure stable inheritance of novel *Wolbachia* infection and (2) control for any immediate or near-immediate effects of the horizontal transfer protocol on fitness.

### Fitness test for *T. kaykai–Wolbachia* interaction

Three fitness traits, total pupae, total offspring and total female offspring, were measured for the 16 *T. kaykai–Wolbachia* lines using a minimum of 35 unmated females from each line. From these measurements pupal survival and offspring sex ratios were derived as proportions. To control for possible *Wolbachia* titer differences associated with age (Jeong & Stouthamer, 2005) among replicates, experimental females were derived from offspring collected on day 1 (the first day) of oviposition by females that were themselves sampled on day 1, i.e., the first-born progeny of first-born progeny. All lines in the final fitness test were assayed at the same time. Pupae were isolated in 12 × 75 mm glass culture tubes. Upon emergence, these isolated females were given eggcards for oviposition and honey for 24 h. Afterwards eggcards were removed and replaced with a fresh eggcard and honey. Each removed eggcard was placed in a culture tube and incubated for 14 days. Each female was given a total six eggcards, the first five for 24 h oviposition, while the sixth eggcard was left with the female for 96 h (4 × 24 h). After the 10-day test period the experiment was stopped. All eggcards were scored for fecundity-offspring reaching pupal development stage, and female offspring production, pupal survival, and offspring sex ratio (female offspring/total offspring). Pupal survival was defined as the ratio between the number of eclosed adult *T. kaykai* and total observed pupal development. Female offspring production, was included as a fecundity measure to illustrate the complexity of offspring production and sex ratio interactions across host and *Wolbachia* types.

### Statistical analysis

The fitness measures were used to test hypotheses related to the effect of host and *Wolbachia* genotype; including host–*Wolbachia* interactions and the effect of horizontal transfer (>97 generations of strict co-inheritance vs. novel combinations created by horizontal transfer). Fligner–Killeen tests were conducted on count data to test statistical assumptions associated with homogeneity of variances for categorical variables (Conover, Johnson & Johnson, 1981).

To partition fitness effects between *T. kaykai* and *Wolbachia* genomes excluding any potential coevolutionary effects associated with the original infection lines, generalized linear models (GLMs) excluding original infections (diagonal cells, Fig. 1) were fit to each fitness measure for all novel *T. kaykai/Wolbachia* combinations (those treatments created by horizontal transfer of *Wolbachia*), with Host and *Wolbachia* as the categorical explanatory variables. For each fitness measure, two GLMs (including the effect of host type and *Wolbachia* type) were fitted to the data, one with and one without host–*Wolbachia* interactions. Analysis of deviance hypothesis tests were then used to determine if any significant differences existed between the two models. Fecundity count data models were assumed to have a quasi-Poisson error structure (Poisson distributed errors corrected for overdispersion) while offspring sex ratio (female offspring/total offspring) and pupal survival (total offspring/total pupae) frequency data models were assumed to have a quasi-binomial error structure (binomial distributed errors corrected for overdispersion) The effects of horizontal transfer on all fitness measures were also analyzed with GLMs using the categorical explanatory variables: novel infections created by horizontal transfer of *Wolbachia* (all off-diagonal cells, Fig. 1) and original infections (all diagonal cells). Nonhomogeneity of fecundity fitness variance was observed for host background, *Wolbachia* background and horizontal transfer (comparing the novel infection lines created by horizontal transfer with the original infection lines), supporting the use of GLMs instead of classical ANOVA (aov) (Table S1).

## Results

### Host fitness variation

**Figure 2:**
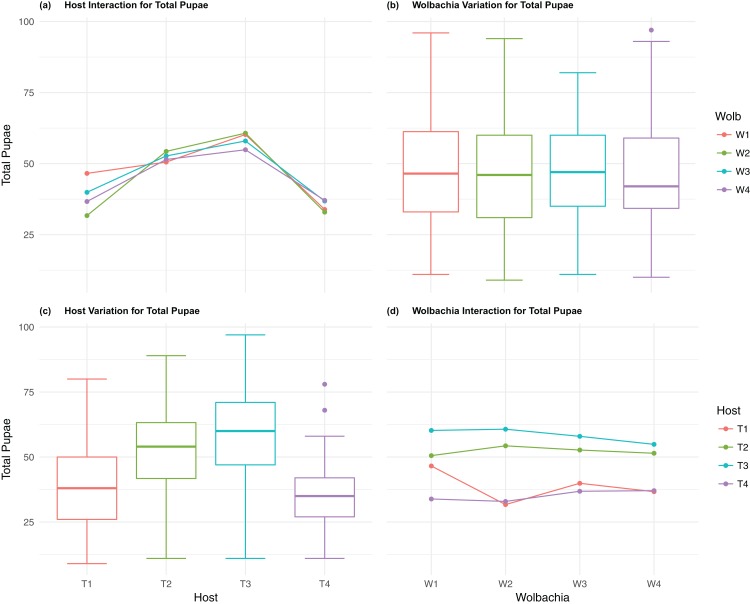
Total Pupae: main effects and two-way interactions. (A) Host Interaction for Total Pupae: variation in total offspring reaching pupal developmental stage across the four *T. kaykai* host strains (*T_1_*–*T_4_*) conditioned on the infected *Wolbachia* types (*W_1_*–*W_4_*) is shown; (B) *Wolbachia* Variation for Total Pupae: box plots show median values and variation across *Wolbachia* types for total pupae. Bold central lines represent median values, box limits represent the interquartile range (*Q_3_*–*Q_1_*), with whisker extensions to data points not more than 1.5× interquartile range. Points outside whiskers are deemed outliers; (C) Host Variation for Total Pupae: box plots show median values and variation across *T. kaykai* host strains for total pupae; (D) *Wolbachia* Interaction for Total Pupae: variation in total offspring reaching pupal developmental stage across the four *Wolbachia* types (*W_1_*–*W_4_*) conditioned on the infected *T. kaykai* host strains (*T_1_*–*T_4_*) is shown.

**Figure 3:**
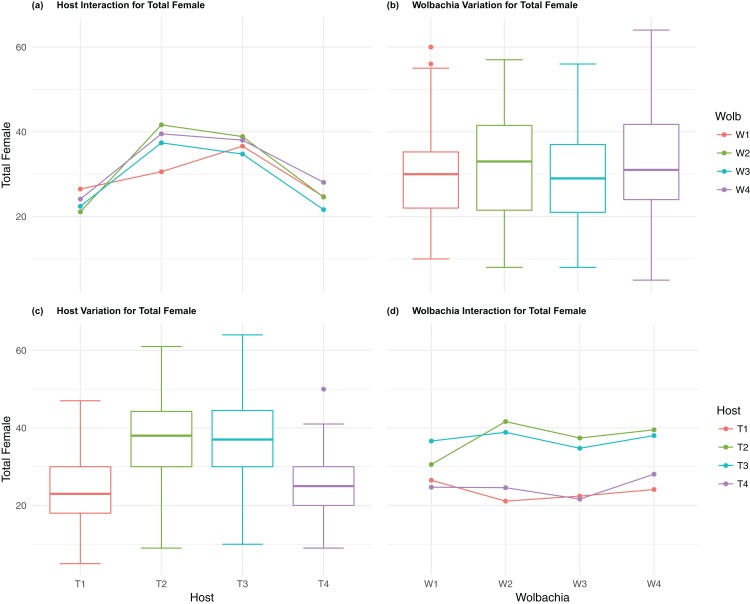
Total Female: main effects and two-way interactions. (A) Host Interaction for Total Female: variation in total female offspring across the four *T. kaykai* host strains (*T_1_*–*T_4_*) conditioned on the infected *Wolbachia* types (*W_1_*–*W_4_*) is shown; (B) *Wolbachia* Variation for Total Female: box plots show median values and variation across *Wolbachia* types for total female offspring. Bold central lines represent median values, box limits represent the interquartile range (*Q_3_*–*Q_1_*), with whisker extensions to data points not more than 1.5× interquartile range. Points outside whiskers are deemed outliers; (C) Host Variation for Total Female: box plots show median values and variation across *T. kaykai* host strains for total female offspring; (D) *Wolbachia* Interaction for Total Female: variation in total female offspring across the four *Wolbachia* types (*W*–*W*) conditioned on the infected *T. kaykai* host strains (*T*–*T*) is shown.

**Figure 4:**
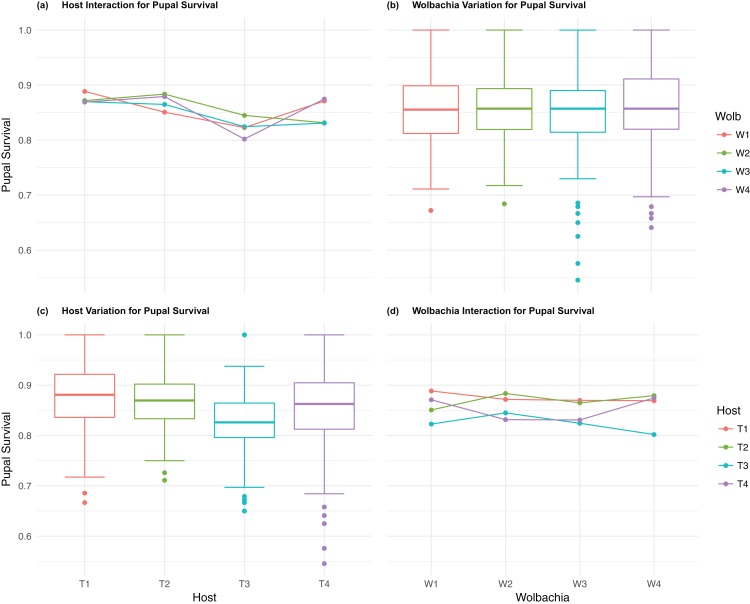
Pupal survival: main effects and two-way interactions. (A) Host Interaction for pupal Survival: variation in survival for offspring reaching pupal developmental stage across the four *T. kaykai* host strains (*T_1_*–*T_4_*) conditioned on the infected *Wolbachia* types (*W_1_*–*W_4_*) is shown; (B) *Wolbachia* Variation for pupal Survival: box plots show median values and variation across *Wolbachia* types for pupal survival. Bold central lines represent median values, box limits represent the interquartile range (*Q_3_*–*Q_1_*), with whisker extensions to data points not more than 1.5× interquartile range. Points outside whiskers are deemed outliers; (C) Host Variation for pupal Survival: box plots show median values and variation across *T. kaykai* host strains for pupal survival; (D) *Wolbachia* Interaction for pupal Survival: variation in survival for offspring reaching pupal developmental stage across the four *Wolbachia* types (*W_1_*–*W_4_*) conditioned on the infected *T. kaykai* host strains (*T_1_*–*T_4_*) is shown.

**Figure 5:**
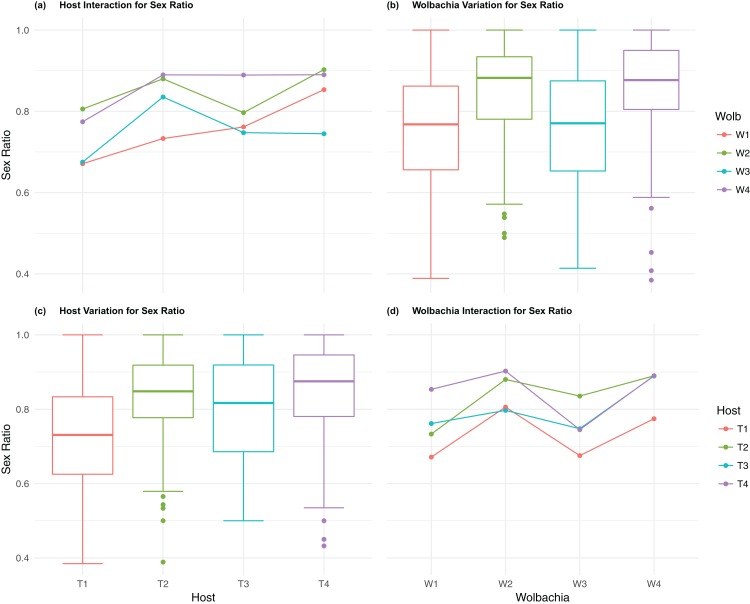
Sex ratio: main effects and two-way interactions. (A) Host Interaction for Sex Ratio: variation in offspring sex ratio (female/total) across the four *T. kaykai* host strains (*T_1_*–*T_4_*) conditioned on the infected *Wolbachia* types (*W_1_*–*W_4_*) is shown; (B) *Wolbachia* Variation for Sex Ratio: box plots show median values and variation across *Wolbachia* types for offspring sex ratio. Bold central lines represent median values, box limits represent the interquartile range (*Q_3_*–*Q_1_*), with whisker extensions to data points not more than 1.5× interquartile range. Points outside whiskers are deemed outliers; (C) Host Variation for Sex Ratio: box plots show median values and variation across *T. kaykai* host strains for offspring sex ratio; (D) *Wolbachia* Interaction for Sex ratio: variation in offspring sex ratio across the four *Wolbachia* types (*W_1_*–*W_4_*) conditioned on the infected *T. kaykai* host strains (*T_1_*–*T_4_*) is shown.

The four host genetic backgrounds used in this experiment showed significant variation for all fitness variables measured (Table 1; Figs. 2–5A). Among the novel horizontal transfer lines (all off-diagonal treatments, Fig. 1) the effect of the host genetic background was significant for fecundity-pupal and female production, survival, and offspring sex ratio (Table 1). The *T_2_* and *T_3_* host lines produced on average about 50% more offspring than the other two host lines (Figs. 2C–3C, Tables S2A and S2B for factorial results). Pupal survival for the least fit host (*T_3_*) was 5.2% lower than for the most fit host (*T_1_*), i.e., 81.7% vs. 86.9% (Fig. 4C, Table S2C). The *T_1_* host line produced fewer female offspring proportionally when compared to the other host lines. Differences in proportional female offspring between host line *T_1_* and the other host lines resulting in offspring sex ratio differences that ranged from 7% to 9% (Fig. 5C, Table S2D). Taken together, relative fitness among the four host lines varied regarding the four measured fitness variables, with host lines *T_2_* and *T_3_* showing overall higher fecundity, and the *T_1_* host line with higher overall survival but lower offspring sex ratio (proportional female offspring).

**Table 1:** Results of generalized linear models (GLMs).

	Fecundity-pupae	Fecundity-female	Pupal survival	Sex ratio
**Intercept**	<0.001***	<0.001***	<0.001***	0.288
**Host (*T_2_*)**	<0.001***	<0.001***	0.020*	0.009**
**Host (*T_3_*)**	<0.001***	<0.001***	<0.001***	<0.001***
**Host (*T_4_*)**	0.354	0.157	0.208	<0.001***
***Wolbachia* (*W_2_*)**	0.603	0.159	0.330	0.001**
***Wolbachia* (*W_3_*)**	0.243	0.276	0.160	0.150
***Wolbachia* (*W_4_*)**	0.605	0.011*	0.084	<0.001***
Host*Wolbachia	0.481	0.061	0.007**	<0.001***

**Notes:**

Generalized linear model results for the four fitness traits evaluated are shown below with significant *p* values for host, *Wolbachia*, and host–*Wolbachia* interaction effects. The significance values in the interactions row refers to the analysis of deviance results comparing GLM models with and without interactions.

### *Wolbachia* fitness variation

The four *Wolbachia* types (*W_1–_W_4_*) had no significant effect on observed variation for pupal fecundity and survival (Table 1; Figs. 2B and 4B). However, a significant *Wolbachia* effect associated with female fecundity was observed (*p* < 0.012, Table 1) with the *W_4_* type, which produced 10.5% more female offspring than the next highest line, *W_1_* (*W_4_* mean = 34.2; *W_1_* mean = 30.6, Table S2B). The influence of *Wolbachia* type was most clearly observed in the highly significant variation among the four types for offspring sex ratio (Fig. 5B; Table 1). The higher number of female offspring produced by *Wolbachia* type *W_4_* was reflected in significant variation for sex ratio with type *W_4_* producing proportionally more female offspring than the next highest line, *W_2_* (*W_4_* sex ratio = 0.85; *W_2_* sex ratio = 0.80), and the two lowest offspring sex ratio *Wolbachia* types, *W_1_* and *W_3_*, producing between 4% and 11% fewer female offspring proportionally than the two highest offspring sex ratio lines (*W_1_* sex ratio = 0.76; *W_3_* sex ratio = 0.74; Table S2D). The fitness effects associated with variation among the *Wolbachia* types was largely confined to significant offspring sex ratio differences, with a residual effect on the overall number of female offspring.

### *Host–Wolbachia* genotype fitness interactions

Fecundity fitness variation, offspring reaching pupal stage of development and female offspring production, for *T. kaykai* was solely a function of the main host and, to a lesser extent, *Wolbachia* type effects, with no significant host–*Wolbachia* interaction (Table 1; Figs. 2A, 3A and 3D). However interaction effects were observed for pupal survival (*p* < 0.007, Figs. 4A and 4D) and strong interaction effects were observed for offspring sex ratio (*p* < 0.001, Table 1; Figs. 5A and 5D). Unlike fecundity, *T. kaykai* fitness regarding pupal survival and offspring sex ratio is a function of strong host effects for both fitness parameters, strong *Wolbachia* effects for offspring sex ratio, and interaction effects associated with distinct host–*Wolbachia* combinations.

### Original versus Novel infection (horizontal transfer effect)

There were no significant effects of horizontal transfer on the number of offspring reaching the pupal stage of development, female offspring production, or offspring sex ratio. Though the mean fecundity values for the original infection lines (diagonal cells, Fig. 1) were higher than the mean values for the horizontal transfer lines (off-diagonal cells), the differences were not significant (Table 2). The only significant effect of horizontal transfer was observed for pupal survival (*p* < 0.05); with horizontal transfer host–*Wolbachia* combinations suffering a small, but consistent, 1.4% decrease in pupal survival relative to the original infection lines (Table 2; Table S2C). Fitness for the novel host–*Wolbachia* combinations created by horizontal transfer was not significantly different from the original combinations, with the exception of a slightly significant negative effect on pupal survival in the horizontally transferred lines.

**Table 2:** Mean fitness values and results of GLMs testing the effect of experimental horizontal transfer.

	Horz. transfer	Original	*p* Value
**Fecundity-pupae**	45.8	49.0	0.098
**Fecundity-female**	30.4	32.7	0.061
**Survival**	0.84	0.86	0.041*
**Sex ratio**	0.79	0.78	0.553

**Notes:**

The mean values and results of GLMs testing the effect of experimental horizontal transfer of *Wolbachia* in *T. kaykai* on fitness traits are shown below. Horz. transfer refers to mean values for experimental treatments created by horizontal transfer of *Wolbachia*. Original refers to mean values for cultures from which horizontally transferred *Wolbachia* were derived.

## Discussion

The fitness variables in which *T. kaykai* and *Wolbachia*’s evolutionary interests would seem to align, fecundity and survival, showed similar patterns of significant host effects, and little or no overall *Wolbachia* effect. There were no significant host–*Wolbachia* interactions associated with fecundity, however a significant interaction effect for survival was observed. The fitness variable that presents a potential conflict of interest for *T. kaykai* and *Wolbachia*, offspring sex ratio, showed significant host, *Wolbachia* and interaction effects.

Taken together, the analysis of main effects on fitness suggests that the absence of any strong variation due to the different host-associated *Wolbachia* strains may be a result of *Wolbachia* genotypes having been selected to the same “good mixer” optimum, in which the influence of *Wolbachia* on host genes optimized for reproductive fitness is attenuated. Combined with significant variation across host types our observations suggest PI-*Wolbachia* essentially “blend into” the fitness landscape of the host environment. This is consistent with expectation given that with respect to reproduction and survival the interests of both the wasp and the *Wolbachia* are aligned since higher fecundity and higher survival favors both parties. Similar results have been observed for *Rickettsia* infections in the whitefly *Bemisia tabaci*, where introgression experiments showed no significant variation among extranuclear genotypes (cytoplasmic elements, including *Rickettsia*) for host fecundity and development fitness measures (Hunter et al., 2016).

In contrast to the host-dependent source of fecundity and survival variation, variation in sex ratio was driven by host, *Wolbachia* and their interaction (Table 2). For symbioses where maternally inherited symbionts manipulate host sex ratios, the interaction between host and symbiont is characterized by conflict over control of offspring sex ratios (Werren, 2011). However the extent to which control is exerted by host and symbiont is often unknown. A recent study on the *B. tabaci–Rickettsia* symbiosis, in which *Rickettsia* manipulate sex ratios in a female-biasing manner, found control over offspring sex ratios was a function of both host genetic background and host–symbiont interactions (Hunter et al., 2016). For infected *T. kaykai* females, offspring sex ratios are a function of the efficiency of *Wolbachia*-induced gamete duplication (the means by which PI-*Wolbachia* converts male offspring to female offspring). *Wolbachia*-nuclear conflict over offspring sex ratios predicts nuclear selection for host resistance to gamete duplication efficiency would favor the production of male offspring in female-biased populations (Stouthamer et al., 2001, 2010). Host nuclear suppression of *Wolbachia*-induced sex ratio distortion has been observed in the butterfly *Hypolimnas bolina* (Hornett et al., 2006), where sex ratio distortion takes the form of male-killing, rather than PI-*Wolbachia* feminization and parthenogenesis-induction. Though previous analysis found no evidence of suppressor alleles in the *T. kaykai* population (Stouthamer et al., 2001), a more detailed analysis of variation for this character in *T. kaykai* may reveal cryptic genetic variation for suppression of gamete duplication. It is possible that the titer of the bacteria may be influenced by the host genome which may, in turn, influence the gamete-duplication ability of *Wolbachia* in a manner similar to the dose-dependent effect of antibiotics on *Wolbachia*-infected *Musicifurax uniraptor* sex ratios (Zchori-Fein, Gottlieb & Coll, 2000). *Wolbachia* titer in *Trichogramma pretiosum* was found to be positively associated with the production of infected female offspring (Lindsey & Stouthamer, 2017a). *Wolbachia* titer is associated with transmission efficiency and may be a target of conflicting sex ratio selection in the *T. kaykai-Wolbachia* symbiosis, with *T. kaykai* selection for less efficiency, resulting more male offspring, particularly in female-biased sex ratio distorted environments.

*Wolbachia* types may vary in their gamete duplication efficiency. Schneider et al. (2013) uncovered cryptic *Wolbachia* population heterogeneity in the European cherry fruit fly, *Rhagoletis cerasi*, when horizontally transferred to novel hosts where subsequent fitness variation in novel host backgrounds was observed. Given the expectation of divergent evolutionary optima for *T. kaykai* and *Wolbachia*, the finding of a significant interaction for offspring sex ratio in our experiment was not surprising. While PI-*Wolbachia* benefits from female-biasing gamete duplication efficiency; *T. kaykai* nuclear genes benefit from gamete duplication inefficiency and male production in female-biased sex ratio distorted populations. The observed significant main and interaction effects for offspring sex ratio may be an indication of cytoplasmic-nuclear genomic conflict over sex ratios in the *T. kaykai–Wolbachia* symbiosis. One proposed resolution to this evolutionary conflict is sex ratio selection on host fertilization frequencies resulting in the loss of female sexual function, the fixation of *Wolbachia* infection, and the alignment of fitness interests favoring the transmission and gamete duplication ability of *Wolbachia* (Stouthamer et al., 2010).

Although the overall effect of horizontal transfer was negative when the original and novel lines were tested for fecundity and survival, with fecundity decreased by 7% (Table S2A and S2B) and pupal survival reduced from 0.86 to 0.84, only survival was shown to be statistically significant (Table 2). It was not possible to distinguish whether these fitness reductions were a result of *Wolbachia* adaptation to host genomic environments subsequently disrupted by horizontal transfer, or simply a result of fitness costs associated with the horizontal transfer procedure used. The 20-generation incubation period for horizontally transferred *Wolbachia* lines in this experiment was intended to control for any immediate fitness effects associated with the horizontal transfer. But it should be noted that negative fitness effects associated with horizontal transfer of *Arsenophonus* inherited symbionts have been observed in aphids (Russell & Moran, 2005), where a four generation horizontal transfer incubation period was used. Horizontal transfer to novel host backgrounds and introgression experiments have shown *Wolbachia* is capable of rapid adaptation to novel host environments (Newton & Sheehan, 2015) and significant phenotypic shifts (Fujii et al., 2001; McGraw et al., 2002; Sasaki, Kubo & Ishikawa, 2002; Jaenike, 2007). The dynamism of host–*Wolbachia* interactions has been exploited in transfection experiments with *Wolbachia* strain *w*MelPop in which transfer to novel hosts has resulting in immune activation that limits competency of cohabiting symbionts like viruses and filarial nematodes (Kambris et al., 2009; Moreira et al., 2009).

The observed pupal survival costs associated with horizontal transfer may be the result of dynamics associated with *T. kaykai–Wolbachia* genomic cooperation (Herre et al., 1999; Rand, Haney & Fry, 2004; Vautrin & Vavre, 2009). In other words, coadaptation between host and *Wolbachia* in the original infected lines may have resulted in reduced survival costs for *T. kaykai*, and such coadaptation may have not had time to occur in the novel host–*Wolbachia* combinations created by horizontal transfer. How *T. kaykai–Wolbachia* coadaptation for pupal survival (or fecundity) would take place is unknown, but it is unlikely in our experiment that changes would take place in the wasp genome since the lines used were 100% homozygous and 100 generations would be too short for an appreciable number of mutations to accumulate. Consequently, changes would have to take place in the *Wolbachia* genome or population. Per wasp egg per generation around 400–1000 *Wolbachia* cells are passed on from mother to her offspring (Stouthamer & Werren, 1993). *Wolbachia* adaptation to a host environment has been observed in the *Wolbachia* strain known as wMelPop when serial passaged in novel host cell culture (McMeniman et al., 2008); an environment not unlike the strict cyto-nuclear inheritance of unmated PI *Wolbachia*-infected species. Newton & Sheehan (2015) found that *Wolbachia* titer adapted to novel *Drosophila melanogaster* nuclear backgrounds in as little as three generations.

Alternatively, *Wolbachia–*mitochondria interactions may play a role in the observed fitness deductions observed for the horizontally transferred lines. Recent results with *T. pretiosum* showed that nuclear introgression of novel genetic background appeared to have no negative fecundity fitness effects (Lindsey & Stouthamer, 2017b). However, horizontal transfer of *Wolbachia* was not possible with *T. pretiosum*, unlike here where *Wolbachia* were experimentally transferred to novel host nuclear and mitochondrial backgrounds. Future experiments in which horizontal transfer and nuclear introgression are applied to the same system would be useful in determining potential *T. kaykai–Wolbachia* coadaptation.

## Conclusion

*Wolbachia* in the *T. kaykai* population would appear to be good “mixers” in regard to shared fitness interests, exhibiting similar mean fitness values across *T. kaykai* backgrounds for fecundity and survival. Though *Wolbachia* may not increase fitness for *T. kaykai* (Russell et al., 2016; Tagami, Miura & Stouthamer, 2001), variation in survival and reproduction among the infected population appears less a function of variation among *Wolbachia,* and more a function of variation among host backgrounds. The strong main and interaction effects observed for offspring sex ratios appear to indicate the good “mixers” phenotype does not apply to this trait, as would be expected for a cytoplasmic sex ratio distorter like PI-*Wolbachia*, and may be a signature of *Wolbachia*-nuclear conflict. Support for the good mixer-evolutionary conflict hypothesis has been demonstrated in introgression experiments with sex ratio-distorting *Rickettsia* infected population of the whitefly, *B. tabaci,* where shared interest traits, such as fecundity, are determined primarily by host genotype alone, while conflicting interest traits, such as offspring sex ratio, are influenced by host–symbiont interactions (Hunter et al., 2016); like the results found with *T. kaykai*. Future nuclear introgression experiments using a similar factorial design would be useful in determining the general applicability of a conflict hypothesis in the *T.kaykai-Wolbachia* symbiosis.

## Supplemental Information

10.7717/peerj.4655/supp-1 Supplemental Information 1 Experimental results used in all analyses. The experimental results for replicates and treatments used for all measured variables are presented.

10.7717/peerj.4655/supp-2 Supplemental Information 2 Fligner-Killeen test results. The results of Fligner-Killeen tests analyzing homogeneity of variances for the categorical variables Host, *Wolbachia*, and horizontal transfer are shown below.

10.7717/peerj.4655/supp-3 Supplemental Information 3 Factorial results for total pupae fecundity. Analysis of the fecundity-total pupae (+/− 1se) of the 4 coevolved experimental lines (shaded) and of the 12 novel combinations of *Wolbachia* (*W*) and host (*T*) (see Fig. 2).

10.7717/peerj.4655/supp-4 Supplemental Information 4 Factorial results for total female offspring fecundity. Analysis of the fecundity-total female offspring (+/− 1se) of the 4 coevolved experimental lines (shaded) and of the 12 novel combinations of *Wolbachia* (*W*) and host (*T*) (see Fig. 3).

10.7717/peerj.4655/supp-5 Supplemental Information 5 Factorial results for pupal survival. Analysis of the pupal survival (+/− 1se) of the four original experimental lines (shaded) and of the 12 novel combinations of *Wolbachia* (*W*) and host (*T*) (see Fig. 4).

10.7717/peerj.4655/supp-6 Supplemental Information 6 Factorial results for ofsspring sex ratio. Analysis of offspring sex ratio (female/total offspring, +1se) of the 4 coevolved experimental lines (shaded) and of the 12 novel combinations of *Wolbachia* (*W*) and host (*T*) (see Fig. 5).

## Additional Information and Declarations

### Field Study Permissions

The following information was supplied relating to field study approvals (i.e., approving body and any reference numbers): Collection permits were obtained from Debra Hughson (science advisor, Mojave National Preserve) and annual reports contributed to the United States National Park Service, Mojave National Preserve.

### Data Availability

The following information was supplied regarding data availability: The raw data used to generate all measured variables are available in the Supplemental Files.

## References

[ref-1] Arakaki N, Miyoshi T, Noda H (2001). *Wolbachia*-mediated parthenogenesis in the predatory thrips *Fanklintothrips vespiformis* (Thysanoptera: Insecta). Proceedings of the Royal Society B: Biological Sciences.

[ref-2] Baldo L, Dunning Hotopp JC, Jolley KA, Bordenstein SR, Biber SA, Choudhury RR, Hayashi C, Maiden MCJ, Tettelin H, Werren JH (2006). Multlocus sequence typing system for the endosymbiont *Wolbachia pipientis*. Applied and Environmental Microbiology.

[ref-3] Conover WJ, Johnson ME, Johnson MM (1981). A comparative study of tests for homogeneity of variances, with applications to the outer continental shelf bidding data. Technometrics.

[ref-6] Fujii Y, Kageyama D, Hoshizaki S, Ishikawa H, Sasaki T (2001). Transfection of *Wolbachia* in Lepidoptera: the feminizer of the adzuki bean borer *Ostrinia scapulalis* causes male killing in the Mediterranean flour moth *Ephestia kuehniella*. Proceedings of the Royal Society B: Biological Sciences.

[ref-7] Herre EA, Knowlton N, Mueller UG, Rehner SA (1999). The evolution of mutualisms: exploring the paths between conflict and cooperation. Trends in Ecology & Evolution.

[ref-8] Hohmann CL, Luck RF, Stouthamer R (2001). Host deprivation effect on reproduction and survival of *Wolbachia*-infected and uninfected *Trichogramma kaykai* (Hymenoptera: Trichogrammatidae). Neotropical Entomology.

[ref-9] Hornett EA, Charlat S, Duplouy AMR, Davies N, Roderick GK, Wedell N, Hurst GDD (2006). Evolution of male-killer suppression in a natural population. PLOS Biology.

[ref-10] Huigens ME (2003). On the Evolution of Wolbachia-Induced Parthenogenesis in Trichogramma wasps.

[ref-11] Huigens ME, Hohmann CL, Luck RF, Gort G, Stouthamer R (2004). Reduced competitive ability due to *Wolbachia* infection in the parasitoid wasp *Trichogramma kaykai*. Entomologia Experimentalis et Applicata.

[ref-12] Huigens ME, Luck RF, Klaassen RHG, Maas MFPM, Timmermans MJTN, Stouthamer R (2000). Infectious parthenogenesis. Nature.

[ref-13] Huigens ME, Stouthamer R, Bourtzis K, Miller TA (2003). Parthenogenesis associated with *Wolbachia*. Insect Symbiosis.

[ref-14] Hunter MS, Asiimwe P, Himler AG, Kelly SE (2016). Host nuclear genotype influences phenotype of a conditional mutualist symbiont. Journal of Evolutionary Biology.

[ref-15] Jaenike J (2007). Spontaneous emergence of a new *Wolbachia* phenotype. Evolution.

[ref-16] Jeong G, Stouthamer R (2005). Genetics of female functional virginity in the parthenogenesis *Wolbachia* infected parasitoid wasp *Telenomus nawai* (Hymenoptera:Scelionidae). Heredity.

[ref-17] Kambris Z, Cook PE, Phuc HK, Sinkins SP (2009). Immune activation by life-shortening *Wolbachia* and reduced filarial competence in mosquitoes. Science.

[ref-18] Kremer N, Charif D, Henri H, Bataille M, Prévost G, Kraaijeveld K, Vavre F (2009). A new case of *Wolbachia* dependence in the genus *Asobara*: evidence for parthenogenesis-induction in *Asobara japonica*. Heredity.

[ref-19] Lindsey ARI, Stouthamer R (2017a). Penetrance of symbiont-mediated parthenogenesis is driven by reproductive rate in a parasitoid wasp. PeerJ.

[ref-20] Lindsey ARI, Stouthamer R (2017b). The effects of outbreeding on a parasitoid wasp fixed for infection with a parthenogenetic-inducing *Wolbachia* symbiont. Heredity.

[ref-21] McGraw EA, Merritt DJ, Droller JN, O’Neill SL (2002). Wolbachia density and virulence attenuation after transfection into a novel host. Proceedings of the National Academy of Sciences of the United States of America.

[ref-22] McMeniman CJ, Lane AM, Fong AWC, Voronin DA, Iturbe-Ormaetxe I, Yamada R, McGraw EA, O’Neill SL (2008). Host adaptation of a *Wolbachia* strain after long-term serial passage in mosquito cell lines. Applied and Environmental Microbiology.

[ref-23] Miura K, Tagami Y (2004). Comparison of life history characters of arrhenotokous and *Wolbachia*-associated thelytokous *Trichogramma kaykai* Pinto and Stouthamer (Hymenoptera: Trichogrammatidae). Annals of the Entomological Society of America.

[ref-24] Moreira LA, Iturbe-Ormaetxe I, Jeffery JA, Lu G, Pyke AT, Hedges LM, Rocha BC, Hall-Mendelin S, Day A, Riegler M, Hugo LE, Johnson KN, Kay BH, McGraw EA, Van den Hurk AF, Ryan PA, O’Neill SL (2009). A *Wolbachia* symbiont in *Aedes aegypti* limits infection with Dengue, Chikungunya, and Plasmodium. Cell.

[ref-25] Newton ILG, Sheehan KB (2015). Passage of *Wolbachia pipientis* through mutant *Drosophila melanogaster* induces phenotypic and genomic changes. Applied and Environmental Microbiology.

[ref-26] Pannebakker BA, Schidlo NS, Boskamp GJF, Dekker L, Van Dooren TJM, Beukeboom LW, Zwaan BJ, Brakefield PM, Van Alphen JJM (2005). Sexual functionality of *Leptipolina clavipes* (Hymenoptera: Figitidae) after reversing *Wolbachia*-induced parthenogenesis. Journal of Evolutionary Biology.

[ref-27] Pfarr KM, Hoerauf A (2007). A niche for *Wolbachia*. Trends in Parasitology.

[ref-28] Pinto JD, Stouthamer R, Platner GR (1997). A new species of Trichogramma (Hymenoptera, Trichogrammatidae) from the Mojave Desert of California as determined by morphological, reproductive, and molecular data. Proceedings of the Entomological Society of Washington.

[ref-29] Rand DM, Haney RA, Fry AJ (2004). Cytonuclear coevolution: the genomics of cooperation. Trends in Ecology & Evolution.

[ref-30] Russell JE (2008). The Ecological and Evolutionary Consequences of Wolbachia Infection in Trichogramma Species.

[ref-31] Russell JA, Moran NA (2005). Horizontal transfer of bacterial symbionts: heritability and fitness effects in a novel aphid host. Applied and Environmental Microbiology.

[ref-32] Russell JE, Saum M, Burgess V, Bollavaram K, Donnell T (2016). Influence of parthenogenesis-inducing *Wolbachia* infection and sexual mode on *Trichogramma kaykai* (Hymenoptera:Trichogrammatidae) fitness. Annals of the Entomological Society of America.

[ref-33] Russell JE, Stouthamer R (2011). The genetics and evolution of obligate reproductive parasitism in *Trichogramma pretiosum* infected with parthenogensis-inducing *Wolbachia*. Heredity.

[ref-34] Sasaki T, Kubo T, Ishikawa H (2002). Interspecific transfer of *Wolbachia* between two lepidopteran insects expressing cytoplasmic incompatibility: a *Wolbachia* variant naturally infecting *Cadra cautella* causes male killing in *Ephestia keuhniella*. Genetics.

[ref-35] Schneider DI, Reigler M, Arthofer W, Mercot H, Stauffer C, Miller WJ (2013). Uncovering *Wolbachia* diversity upon artificial host transfer. PLOS ONE.

[ref-36] Stouthamer R, Kazmer DJ (1994). Cytogenetics of microbe-associated parthenogenesis and its consequences for gene flow in *Trichogramma* wasps. Heredity.

[ref-37] Stouthamer R, Luck RF, Hamilton WD (1990). Antibiotics cause parthenogenetic *Trichogramma* (Hymenoptera, Trichogrammatidae) to revert to sex. Proceedings of the National Academy of Sciences of the United States of America.

[ref-38] Stouthamer R, Russell JE, Vavre F, Nunney L (2010). Intragenomic conflict in populations infected by parthenogenesis inducing *Wolbachia* ends with irreversible loss of sexual reproduction. BMC Evolutionary Biology.

[ref-39] Stouthamer R, Van Tilborg M, De Jong JH, Nunney L, Luck RF (2001). Selfish element maintains sex in natural populations of a parasitoid wasp. Proceedings of the Royal Society B: Biological Sciences.

[ref-40] Stouthamer R, Werren JH (1993). Microbes associated with parthenogenesis in wasps of the genus *Trichogramma*. Journal of Invertebrate Pathology.

[ref-41] Tagami Y, Miura K, Stouthamer R (2001). How does infection with parthenogenesis-inducing *Wolbachia* reduce the fitness of Trichogramma?. Journal of Invertebrate Pathology.

[ref-42] Tagami Y, Miura K, Stouthamer R (2002). Positive effect of fertilization on the survival rate of immature stages in a *Wolbachia*-associated thelytokous line of *Trichogramma deion* and *T. kaykai*. Entomologia Experimentalis et Applicata.

[ref-43] Turelli M (1994). Evolution of incompatibility-inducing microbes and their hosts. Evolution.

[ref-44] Vautrin E, Vavre F (2009). Interactions between vertically transmitted symbionts: cooperation or conflict?. Trends in Microbiology.

[ref-45] Wade MJ, Goodnight CJ (2006). Cyto-nuclear epistasis: two-locus random genetic drift in hermaphroditic and dioecious species. Evolution.

[ref-46] Weeks AR, Breeuwer JAJ (2001). Wolbachia-induced parthenogenesis in a genus of phytophagous mites. Proceedings of the Royal Society B: Biological Sciences.

[ref-47] Weeks AR, Turelli M, Harcombe WR, Reynolds KT, Hoffmann AA (2007). From parasite to mutualist: rapid evolution of *Wolbachia* in natural populations of *Drosophila*. PLOS Biology.

[ref-48] Werren JH (1997). Biology of *Wolbachia*. Annual Review of Entomology.

[ref-49] Werren JH (2011). Selfish genetic elements, genetic conflict, and evolutionary innovation. Proceedings of the National Academy of Sciences of the United States of America.

[ref-50] Werren JH, Windsor D, Guo LR (1995). Distribution of *Wolbachia* among neotropical arthropods. Proceedings of the Royal Society B: Biological Sciences.

[ref-51] Zchori-Fein E, Gottlieb Y, Coll M (2000). *Wolbachia* Density and Host Fitness Components in *Muscidifurax uniraptor* (Hymenoptera: Pteromalidae). Journal of Invertebrate Pathology.

