# Supplementary material for: Host and symbiont genetic contributions to fitness in a Trichogramma–Wolbachia symbiosis
Source: PeerJ. 2018 Apr 19;6:e4655. doi: 10.7717/peerj.4655 (PMC5911386; doi:10.7717/peerj.4655)
Supplement: Supplemental Information 2 — The results of Fligner-Killeen tests analyzing homogeneity of variances for the categorical variables Host, Wolbachia, and horizontal transfer are shown below. [file peerj-06-4655-s002.docx]

**Supp. Table 1**. The results of Fligner-Killeen tests analyzing homogeneity of variances for the categorical variables Host, *Wolbachia*, and horizontal transfer are shown below.

| **Fecundity-pupae** | ***χ^2^*** | **df** | **p-value** |
| --- | --- | --- | --- |
| **Host** | 3.839 | 3 | 0.279 |
| ***Wolbachia*** | 22.746 | 3 | <0.001*** |
| **Horz. transfer** | 5.997 | 1 | 0.014* |
| **Fecundity-female** | ***χ^2^*** | **df** | **p-value** |
| **Host** | 10.710 | 3 | 0.013* |
| ***Wolbachia*** | 17.791 | 3 | <0.001*** |
| **Horz. transfer** | 7.075 | 1 | 0.007** |
