# Supplementary material for: Host and symbiont genetic contributions to fitness in a Trichogramma–Wolbachia symbiosis
Source: PeerJ. 2018 Apr 19;6:e4655. doi: 10.7717/peerj.4655 (PMC5911386; doi:10.7717/peerj.4655)
Supplement: Supplemental Information 3 — Analysis of the fecundity-total pupae (+/− 1se) of the 4 coevolved experimental lines (shaded) and of the 12 novel combinations of Wolbachia (W) and host (T) (see Fig. 2). [file peerj-06-4655-s003.docx]

**Supp. Table 2a**. Analysis of the fecundity-total pupae (+/- 1se) of the 4 coevolved experimental lines (shaded) and of the 12 novel combinations of *Wolbachia* (*W*) and host (*T*) (see Figure 2).

|  | ***T_1_*** | ***T_2_*** | ***T_3_*** | ***T_4_*** | ***_Total (novel)_ ^1^*** | ***_Total_^2^*** |
| --- | --- | --- | --- | --- | --- | --- |
| ***W_1_*** | 46.6 (3.1) | 50.5 (2.9) | 60.2 (3.5) | 33.9 (1.8) | *48.1 (2.0)* | *47.8 (1.7)* |
| ***W_2_*** | 31.7 (3.0) | 54.3 (2.5) | 60.7 (3.8) | 32.9 (1.8) | *43.1 (2.3)* | *46.0 (1.9)* |
| ***W_3_*** | 39.9 (3.1) | 52.7 (2.4) | 58.0 (2.3) | 36.8 (2.9) | *43.4 (1.7)* | *46.9 (1.5)* |
| ***W_4_*** | 36.7 (2.7) | 51.5 (3.0) | 54.9 (3.6) | 37.1 (2.2) | *48.1 (2.0)* | *45.5 (1.7)* |
| ***_Total N(O)_^1^*** | *36.4(1.7)* | *51.6 (1.6)* | *58.5 (2.1)* | *34.6 (1.3)* | ***45.8(49.0)*** |  |
| ***_Total_^2^*** | *39.1 (1.6)* | *52.2 (1.4)* | *58.4 (1.7)* | *35.2 (1.1)* |  | ***46.6*** |

1. *_Total(novel)_* are the means of the novel (non-coevolved) Host-*Wolbachia* combinations for host (columns) and *Wolbachia* (rows). The grand mean value (bold) and the grand mean value for the coevolved lines (bold, in parentheses and shaded) are also given.

*2. _Total_* are the column and row means of all Host-*Wolbachia* combinations. The grand mean value is in bold.
