# Supplementary material for: Host and symbiont genetic contributions to fitness in a Trichogramma–Wolbachia symbiosis
Source: PeerJ. 2018 Apr 19;6:e4655. doi: 10.7717/peerj.4655 (PMC5911386; doi:10.7717/peerj.4655)
Supplement: Supplemental Information 4 — Analysis of the fecundity-total female offspring (+/− 1se) of the 4 coevolved experimental lines (shaded) and of the 12 novel combinations of Wolbachia (W) and host (T) (see Fig. 3). [file peerj-06-4655-s004.docx]

**Supp. Table 2b**. Analysis of the fecundity-total female offspring (+/- 1se) of the 4 coevolved experimental lines (shaded) and of the 12 novel combinations of *Wolbachia* (*W*) and host (*T*) (see Figure 3).

|  | ***T_1_*** | ***T_2_*** | ***T_3_*** | ***T_4_*** | ***_Total (novel)_ ^1^*** | ***_Total_^2^*** |
| --- | --- | --- | --- | --- | --- | --- |
| ***W_1_*** | 26.5 (1.5) | 30.6 (1.7) | 36.6 (2.1) | 24.7 (1.1) | *30.6 (1.1)* | *29.6 (0.9)* |
| ***W_2_*** | 21.1 (1.8) | 41.6 (1.8) | 38.9 (2.1) | 24.6 (1.4) | *29.0 (1.3)* | *32.3 (1.2)* |
| ***W_3_*** | 22.4 (1.6) | 37.4 (1.5) | 34.8 (1.1) | 21.6 (1.6) | *27.4 (1.2)* | *29.2 (1.0)* |
| ***W_4_*** | 24.1 (1.8) | 39.5 (2.2) | 38.0 (2.2) | 28.1 (1.4) | *34.2 (1.4)* | *32.8 (1.1)* |
| ***_Total N(O)_^1^*** | *22.6(1.0)* | *35.8 (1.1)* | *37.9 (1.2)* | *23.6 (0.8)* | ***30.4(32.7)*** |  |
| ***_Total_^2^*** | *23.7 (0.8)* | *37.1 (1.0)* | *37.1 (1.0)* | *24.7 (0.7)* |  | ***30.9*** |

1. *_Total(novel)_* are the means of the novel (non-coevolved) Host-*Wolbachia* combinations for host (columns) and *Wolbachia* (rows). The grand mean value (bold) and the grand mean value for the coevolved lines (bold, in parentheses and shaded) are also given.

*2. _Total_* are the column and row means of all Host-*Wolbachia* combinations. The grand mean value is in bold.
