# Supplementary material for: Host and symbiont genetic contributions to fitness in a Trichogramma–Wolbachia symbiosis
Source: PeerJ. 2018 Apr 19;6:e4655. doi: 10.7717/peerj.4655 (PMC5911386; doi:10.7717/peerj.4655)
Supplement: Supplemental Information 6 — Analysis of offspring sex ratio (female/total offspring, +1se) of the 4 coevolved experimental lines (shaded) and of the 12 novel combinations of Wolbachia (W) and host (T) (see Fig. 5). [file peerj-06-4655-s006.docx]

**Supp. Table 2d.** Analysis of offspring sex ratio (female/total offspring, +1se) of the 4 coevolved experimental lines (shaded) and of the 12 novel combinations of *Wolbachia* (*W*) and host (*T*) (see Figure 5).

|  | ***T_1_*** | ***T_2_*** | ***T_3_*** | ***T_4_*** | ***_Total (novel)_^1^*** | ***_Total_^2^*** |
| --- | --- | --- | --- | --- | --- | --- |
| ***W_1_*** | 0.65 (.09) | 0.71 (.08) | 0.74 (.08) | 0.84 (.07) | *0.76 (.04)* | *0.73 (.04)* |
| ***W_2_*** | 0.76 (.09) | 0.87 (.06) | 0.76 (.08) | 0.90 (.06) | *0.80 (.04)* | *0.82 (.04)* |
| ***W_3_*** | 0.65 (.09) | 0.83 (.07) | 0.73 (.08) | 0.71 (.08) | *0.74 (.05)* | *0.74 (.04)* |
| ***W_4_*** | 0.75 (.08) | 0.88 (.06) | 0.87 (.06) | 0.88 (.06) | *0.85 (.04)* | *0.85 (.03)* |
| ***_Total N(O)_^1^*** | *0.72 (.05)* | *0.81 (.04)* | *0.79 (.04)* | *0.81 (.04)* | ***0.79(0.78)*** |  |
| ***_Total_^2^*** | *0.69 (.04)* | *0.82 (.03)* | *0.78 (.04)* | *0.83 (.03)* |  | ***0.78*** |

*1.* *_Total(novel)_* are the proportions of the novel (non-coevolved) Host-*Wolbachia* combinations for host (columns) and *Wolbachia* (rows). The overall proportion values for the novel infections (bold) and for the coevolved lines (bold, in parentheses and shaded) are also given. *2. _Total_* are the column and row proportions of all Host-*Wolbachia* combinations. The overall proportion value is in bold.
